# Fish and Shellfish As the Dominant Dietary Sources of Vitamin D Among Japanese Adults: National Surveys (2015-2024)

**DOI:** 10.7759/cureus.119672

**Published:** 2026-11-14

**Authors:** Takuya Omura

**Affiliations:** 1 Department of Metabolic Research, Research Institute, National Center for Geriatrics and Gerontology, Obu, JPN; 2 Department of Diabetes and Endocrinology, National Center for Geriatrics and Gerontology, Obu, JPN

**Keywords:** food sources, japan, national health and nutrition survey, seafood, vitamin b12, vitamin d

## Abstract

Background: Fish and shellfish provide most dietary vitamin D in Japan, yet their consumption has declined. This study examined their contribution to dietary vitamin D among Japanese adults and compared published mean contributions from fish and shellfish and other foods. Vitamin B12 and protein were examined as comparator nutrients.

Methods: Published estimates for adults aged 20 years or older were analyzed from eight National Health and Nutrition Surveys (2015-2019 and 2022-2024). Principal comparisons were restricted to routine surveys using matching food composition tables (2015 versus 2017 and 2019 versus 2023). Contributions from other foods were calculated by subtracting the fish and shellfish contribution from the published food-group total. No inferential tests were performed.

Results: Fish and shellfish supplied 75-78% of mean dietary vitamin D in all seven surveys with adult-specific food-source data. In 2019 and 2023, median fish and shellfish intake was 55.0 and 45.7 g/day (-16.9%), and median vitamin D intake was 3.6 and 3.1 μg/day (-13.9%). Mean ± standard deviation (SD) vitamin D intake was 7.2 ± 8.9 and 6.5 ± 7.9 μg/day, of which fish and shellfish supplied 5.6 and 5.0 μg/day and other foods 1.6 and 1.5 μg/day; allowing for rounding, any increase from other foods (at most 0.1 μg/day) was much smaller than the decrease from fish and shellfish (0.5-0.7 μg/day). For vitamin B12 (6.5 ± 7.9 and 5.5 ± 5.6 μg/day), published contributions from other foods showed no apparent increase (2.1 and 2.0 μg/day), whereas total protein intake changed little (72.2 ± 25.0 and 71.3 ± 24.1 g/day). Food-source contributions are published as means without SDs. In 2019, fish and shellfish supplied 78% of mean vitamin D and 68% of mean vitamin B12 but only 18% of mean protein. In 2023, median vitamin D intake was 2.1 μg/day at ages 20-29 years and 4.2 μg/day at ages 75 years or older.

Conclusions: Fish and shellfish remained the dominant source of dietary vitamin D among Japanese adults. Between 2019 and 2023, lower published mean contributions of vitamin D and vitamin B12 from fish and shellfish were not accompanied by an apparent increase from other foods, whereas total protein intake changed little. This nutrient-specific contrast suggests that changes in micronutrient supply may not be reflected in total protein intake. The aggregate data cannot establish individual food substitutions or changes in serum 25-hydroxyvitamin D.

## Introduction

Fish and shellfish have long been the leading dietary source of vitamin D in Japan, as shown by an analysis of the 2013 National Health and Nutrition Survey (NHNS) [1] and a smaller Japanese study [2]. At the same time, national fisheries statistics document a long-term decline in seafood consumption [3]. When a dominant food source declines, the nutritional consequences depend on whether other foods supply the same nutrients. Meat, for example, may replace fish as a source of protein but not necessarily as a source of vitamin D.

Low serum 25-hydroxyvitamin D (25(OH)D) has been reported in selected Japanese populations, including young women [4] and urban health-checkup attendees [5,6], although these studies do not provide national prevalence estimates. Criteria for interpreting serum 25(OH)D concentrations differ between guidelines, which address different populations and purposes [7,8]. Serum 25(OH)D also depends on cutaneous synthesis and cannot be inferred from dietary intake alone [2]. Food-source data nevertheless show where the dietary supply of vitamin D is concentrated and, therefore, where it may be vulnerable.

Using published NHNS estimates from 2015 to 2024, the primary objective was (1) to describe the contribution of fish and shellfish to mean dietary vitamin D intake across surveys. The secondary objectives were (2) to compare published mean nutrient contributions from fish and shellfish and other foods between 2019 and 2023, using vitamin B12 and protein as comparator nutrients; (3) to compare the nutrient contributions per 100 g of fish and shellfish versus meat; and (4) to describe age-group differences in 2023. The descriptive expectation was that lower fish and shellfish contributions would coincide with lower vitamin D and vitamin B12 intakes but smaller differences in total protein intake, without an apparent increase in contributions from other foods. This expectation was not tested statistically. The aggregate estimates cannot identify individual food substitutions, changes in serum vitamin D status, or causal effects.

## Materials and methods

Data source and participants

Estimates were extracted from the NHNS reports for 2015-2019 and 2022-2024 [9-17]; no surveys were conducted in 2020-2021. The NHNS uses a one-day, semi-weighed household dietary record, with consumption allocated among household members. For adults aged 20 years or older, the sample size, mean, standard deviation (SD), and median were extracted for vitamin D and fish and shellfish intake, as were the mean and SD for total vitamin B12 and protein intake. Values are presented as mean ± SD or median. The food-group tables report mean contributions without corresponding SDs. Adult-specific food-source estimates were available for 2016-2019 and 2022-2024; the 2015 food-source table included people aged one year or older. The analysis used published aggregate estimates; the author had no access to individual records and did not apply or recalculate survey weights. The source tables, reported values, and calculation rules are detailed in the Appendices.

Comparability

The surveys used a common population-based sampling framework and one-day semi-weighed household dietary records, although the selected districts, participation, and sample composition varied. Estimates in this analysis consistently refer to adults aged 20 years or older. The expanded 2016 survey initially selected 475 districts, of which 13 were excluded because of natural disasters; the expanded 2024 survey selected 473 districts, with eight rather than 10 districts in Ishikawa Prefecture after the January 2024 Noto Peninsula earthquake. For national estimates, both expanded surveys applied prefecture-based weights to adjust for differences in participating household numbers relative to routine surveys. The 2016 weights used the preceding three survey years and the 2024 weights used 2022 and 2023. These weights did not standardize the results to the national sex or age structure. The author used all published estimates without additional weighting or age- or sex-standardization.

The 2015-2017 NHNS used the 2010 food composition tables. The 2018 survey used the Seventh Revised (2015) tables with supplementary editions through 2017, and the 2019 and 2022-2024 surveys used supplementary editions through 2018. Principal paired comparisons were therefore made between routine surveys in 2015 and 2017 and in 2019 and 2023, which used matching tables within each pair. The 2019 survey was the last routine survey before the 2020-2021 interruption, and 2023 was the latest routine survey in the analyzed period. The expanded surveys were retained in the descriptive series, but not in the paired comparisons; 2022 was also retained in the series and was not selected as an endpoint.

Descriptive calculations

Medians were rounded to one decimal place before plotting, labeling, and computing relative differences. Percentage change was calculated as $\frac{\mathrm{Later} - \mathrm{Earlier}}{\mathrm{Earlier}} \times 100\%$, using the published values at the precision shown in the text. The contribution of fish and shellfish to each nutrient was calculated by dividing its mean contribution by the corresponding published mean total in the food-group table; the percentage was rounded to the nearest whole number. These are ratios of group means, not means of individual percentages. The published values were used at their reported precision. Where the same total was reported at different precision in different tables, the food-group table was used for the food-source denominator.

For 2019 and 2023, vitamin D from other foods was calculated as the total minus the fish and shellfish contribution, using published rounded means. The same approach was applied to protein and vitamin B12, and the share of each nutrient supplied by fish and shellfish was calculated. Amounts per 100 g of the food group consumed were calculated by dividing the group's mean nutrient contribution by its mean intake weight and multiplying by 100. Mean contributions from meat, eggs, and mushrooms were also extracted, as were 2023 age-specific medians for ages 20-29 and ≥75 years. These values describe food groups, not individual foods, and do not establish causal contributions to changes in total intake. Calculated residuals and between-survey differences are sensitive to rounding of the published means. For a rounding sensitivity check, each mean reported to one decimal place was treated as lying within 0.05 units of its displayed value. Ranges derived from these bounds are arithmetic rounding ranges, not confidence intervals. The 2019-2023 decrease in the fish and shellfish contribution was also expressed as a percentage of the 2019 mean total for each nutrient.

Statistical considerations and ethics

Only descriptive comparisons were conducted. The published aggregates do not provide the survey-design variance estimates needed for valid between-survey inference; therefore, no hypothesis tests, p-values, or test statistics are reported. One-day records do not measure usual individual intake, and vitamin D supplements were not separately quantified. Reporting was informed by the Strengthening the Reporting of Observational Studies in Epidemiology (STROBE) statement and its nutritional epidemiology extension (STROBE-nut) [18,19]. Because only publicly available, aggregated, non-identifiable data were analyzed, ethical review and informed consent were not required.

## Results

Survey estimates

Table 1 summarizes the eight surveys.

**Table 1:** National Survey Estimates for Japanese Adults Aged 20 Years or Older Values are mean ± SD or median for adults aged 20 years or older, taken from the published NHNS tables. *Food composition tables: 2010 edition, or the Seventh Revised (2015) edition with supplementary editions through 2017 (S16-17) or 2018 (S16-18). ^†^Percentage of mean vitamin D supplied by fish and shellfish, calculated by the author from food-group means rounded to one decimal place; it is a ratio of group means, not a mean of individual percentages. -: not shown because the 2015 food-source table covered people aged one year or older. In 2018, the food-group total (6.9 μg/day) was used as the denominator; the age-group table reported 6.95 μg/day. Expanded surveys (2016 and 2024) used prefecture-based weighting. No statistical tests were performed. NHNS: National Health and Nutrition Survey; SD: standard deviation

Year	Survey type	Food composition table*	n	Vitamin D, mean ± SD (μg/day)	Vitamin D, median (μg/day)	Fish and shellfish, mean ± SD (g/day)	Fish and shellfish, median (g/day)	Vitamin D from fish and shellfish (%)^†^
2015	Routine	2010	6,172	7.9 ± 9.0	4.5	74.2 ± 71.4	60.8	-
2016	Expanded	2010	21,851	7.8 ± 8.2	4.1	70.5 ± 65.2	58.0	78
2017	Routine	2010	5,794	7.3 ± 8.2	4.0	68.8 ± 69.2	55.0	75
2018	Routine	2015 + S16-17	5,743	7.0 ± 8.1	3.8	70.1 ± 68.8	59.0	78
2019	Routine	2015 + S16-18	4,927	7.2 ± 8.9	3.6	68.5 ± 71.5	55.0	78
2022	Routine	2015 + S16-18	4,453	6.5 ± 8.0	3.2	61.4 ± 67.7	42.8	75
2023	Routine	2015 + S16-18	4,472	6.5 ± 7.9	3.1	62.6 ± 67.8	45.7	77
2024	Expanded	2015 + S16-18	15,716	6.9 ± 6.5	3.3	63.0 ± 51.2	46.7	77

Mean vitamin D intake was approximately 1.8-2.1 times the median, consistent with right-skewed intake distributions. In the comparable routine surveys of 2015 and 2017, median vitamin D intake was 4.5 and 4.0 μg/day (-11.1%), and median fish and shellfish intake was 60.8 and 55.0 g/day (-9.5%). In 2019 and 2023, the corresponding medians were 3.6 and 3.1 μg/day (-13.9%) and 55.0 and 45.7 g/day (-16.9%) (Figure 1).

**Figure 1:**
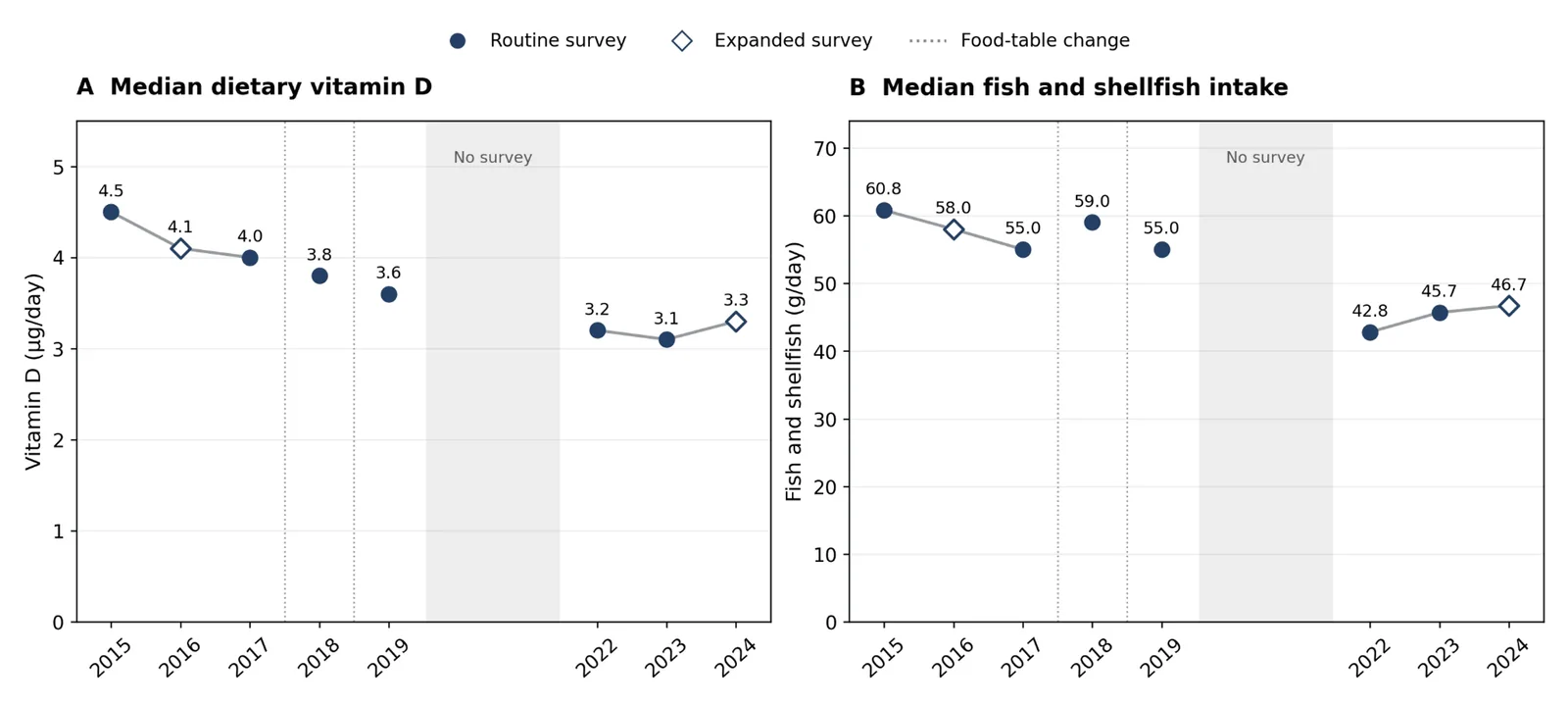
Median Dietary Vitamin D and Fish and Shellfish Intakes Among Japanese Adults, 2015-2024 Panel A: median dietary vitamin D (μg/day); Panel B: median fish and shellfish intake (g/day) among adults aged 20 years or older. Values are published medians rounded to one decimal place. The paired relative differences stated in the text are calculated from rounded medians for 2015-2017 and 2019-2023; no statistical tests were performed. Filled circles: routine surveys; open diamonds: expanded surveys. Shading marks 2020-2021, when no surveys were conducted, and dotted vertical lines separate food composition table versions. Both vertical axes start at zero.

Mean ± SD intakes in 2019 and 2023 were 7.2 ± 8.9 and 6.5 ± 7.9 μg/day for vitamin D (-9.7%) and 68.5 ± 71.5 and 62.6 ± 67.8 g/day for fish and shellfish (-8.6%). The series was not monotonic: fish and shellfish intake was higher in 2023 than in 2022. In the expanded 2024 survey, the medians were 3.3 μg/day and 46.7 g/day.

Dominance of fish-derived vitamin D

Fish and shellfish accounted for 75-78% of mean dietary vitamin D in each of the seven surveys with adult-specific food-group estimates: 78% in 2016, 75% in 2017, 78% in 2018, 78% in 2019, 75% in 2022, 77% in 2023, and 77% in 2024 (Table 1). The 2015 value of 77% referred to people aged one year or older and was therefore excluded from adult-specific comparisons.

Contributions from fish and other foods

Food-source contributions are based on means rather than medians. In 2019 and 2023, fish and shellfish supplied 5.6 and 5.0 μg/day of mean dietary vitamin D, whereas all other foods combined supplied 1.6 and 1.5 μg/day (Table 2 and Figure 2).

**Table 2:** Mean Intake of Vitamin D, Vitamin B12, and Protein by Food Source, 2019 Versus 2023 Mean daily intake among adults aged 20 years or older in the 2019 and 2023 NHNS food-group tables, which report means without SDs. 'All other foods' was calculated by the author as total minus fish and shellfish; rows labeled 'of which' are subsets already included in 'All other foods'. Values are group means and do not describe within-person substitution. No statistical tests were performed. NHNS: National Health and Nutrition Survey; SD: standard deviation

Nutrient	Food source	2019	2023
Vitamin D (μg/day)	Total	7.2	6.5
	Fish and shellfish	5.6	5.0
	All other foods (combined)	1.6	1.5
	Of which: meat	0.2	0.2
	Of which: eggs	0.7	0.7
	Of which: mushrooms	0.2	0.2
Vitamin B12 (μg/day)	Total	6.5	5.5
	Fish and shellfish	4.4	3.5
	All other foods (combined)	2.1	2.0
	Of which: meat	0.9	0.8
Protein (g/day)	Total	72.2	71.3
	Fish and shellfish	13.1	12.0
	All other foods (combined)	59.1	59.3
	Of which: meat	17.3	18.2

**Figure 2:**
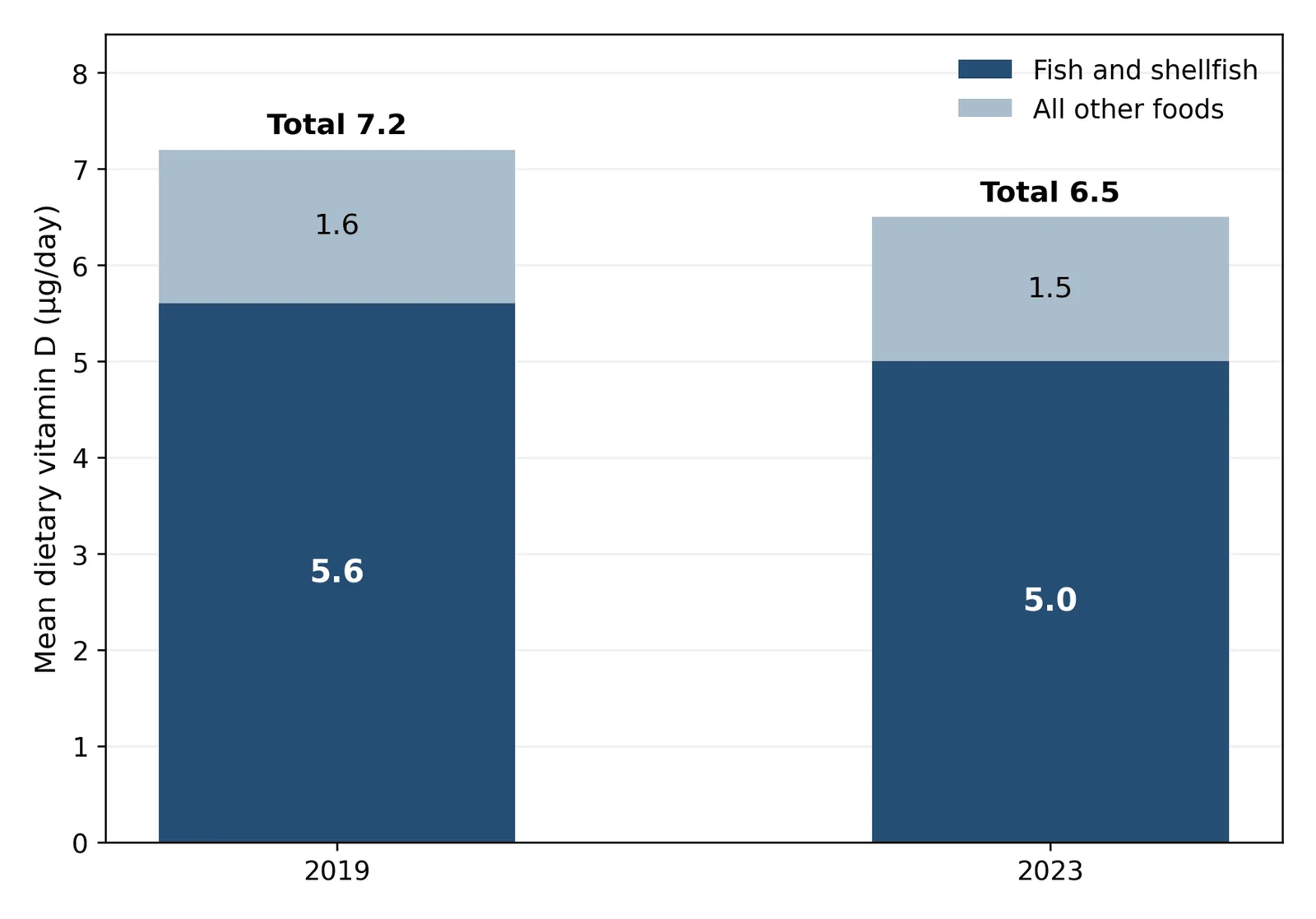
Mean Dietary Vitamin D From Fish and Shellfish and From All Other Foods, 2019 and 2023 Stacked bars show mean vitamin D (μg/day) among adults aged 20 years or older in the 2019 and 2023 NHNS food-group tables: the published contribution from fish and shellfish and the contribution from all other foods, calculated by the author as total minus fish and shellfish. Values are rounded to 0.1 μg/day. No statistical tests were performed because SDs are not published for these values. The bars do not describe within-person substitution. NHNS: National Health and Nutrition Survey; SD: standard deviation

Of the 0.7-μg/day difference in the published mean total, 0.6 μg/day was in the fish and shellfish contribution. Allowing for rounding to 0.1 μg/day, the difference (2023 minus 2019) in the contribution from all other foods could range from approximately -0.3 to +0.1 μg/day, while the fish and shellfish contribution was lower by approximately 0.5-0.7 μg/day. These are arithmetic bounds, not confidence intervals or evidence of individual food substitution. Eggs supplied 0.7 μg/day and mushrooms 0.2 μg/day in both surveys.

Vitamin B12 and protein

Mean ± SD vitamin B12 intake was 6.5 ± 7.9 μg/day in 2019 and 5.5 ± 5.6 μg/day in 2023; fish and shellfish supplied 4.4 and 3.5 μg/day, and all other foods 2.1 and 2.0 μg/day; allowing for rounding, the corresponding ranges were approximately -0.3 to +0.1 μg/day for other foods and a decrease of 0.8-1.0 μg/day for fish and shellfish. In contrast, mean ± SD total protein intake was 72.2 ± 25.0 and 71.3 ± 24.1 g/day. Protein from fish and shellfish was 13.1 and 12.0 g/day, whereas protein from meat was 17.3 and 18.2 g/day (Table 2). SDs are not published for these food-group contributions. Fish and shellfish therefore supplied 78% and 77% of mean vitamin D, 68% and 64% of mean vitamin B12, and 18% and 17% of mean protein in 2019 and 2023. The 2019-2023 decrease in the fish and shellfish contribution corresponded to 8.3% of the 2019 mean total for vitamin D, 13.8% for vitamin B12, and 1.5% for protein. In 2019, per 100 g of each food group consumed (mean intakes: fish and shellfish, 68.5 g/day; meat, 101.0 g/day), fish and shellfish versus meat provided 19.1 versus 17.1 g of protein, 8.2 versus 0.2 μg of vitamin D, and 6.4 versus 0.9 μg of vitamin B12.

Age groups

In 2023, median vitamin D intake was 2.1 μg/day among adults aged 20-29 years and 4.2 μg/day among those aged 75 years or older; median fish and shellfish intake was 15.0 and 68.7 g/day, respectively. These cross-sectional estimates were not age- or sex-standardized and cannot separate age from cohort effects.

## Discussion

Across national surveys from 2016 to 2024, fish and shellfish consistently supplied about three-quarters of the mean dietary vitamin D among Japanese adults. In the comparable 2019 and 2023 surveys, fish-derived vitamin D was lower (5.6 versus 5.0 μg/day), whereas vitamin D from all other foods combined was essentially unchanged (1.6 versus 1.5 μg/day). Vitamin B12 showed a similar pattern, but total protein intake changed little. At the aggregate level, published contributions from other foods showed no apparent increase at the reported precision. These aggregate estimates cannot establish whether individual food substitutions occurred.

These contrasts reflect how concentrated each nutrient's supply was in fish and shellfish. Vitamin B12 is ordinarily supplied by animal-derived foods, including fish and shellfish [20]. In 2019, fish and shellfish supplied 78% of mean vitamin D and 68% of mean vitamin B12 but only 18% of mean protein. Consequently, the lower fish-derived supply corresponded to 8.3% of mean total vitamin D and 13.8% of mean total vitamin B12, but only 1.5% of mean total protein. Per 100 g consumed, they provided more than 30 times as much vitamin D and approximately seven times as much vitamin B12 as meat, but a similar amount of protein. These differences suggest that replacing fish and shellfish with meat could preserve some protein intake while providing less vitamin D and vitamin B12, depending on the foods and quantities consumed. Monitoring based on total protein or broad animal-food intake may miss such changes in micronutrients whose supply is concentrated in a single food group.

The age gradient is also notable. In 2023, median fish and shellfish intake among adults aged 20-29 years was less than one-quarter of that among adults aged 75 years or older, and their median vitamin D intake was half. These cross-sectional differences may reflect age-related changes, cohort-specific dietary patterns, or both; these possibilities cannot be distinguished using the available data.

Dietary intake is only one determinant of vitamin D status. Serum 25(OH)D also reflects ultraviolet (UV)-dependent cutaneous synthesis, and few foods are naturally rich in vitamin D [21]. In 107 adults from two Japanese regions, Asakura and colleagues found an association between dietary vitamin D and serum 25(OH)D3 after accounting for measured UV exposure [2]. A meta-analysis of randomized trials found that fish intake could modestly increase serum 25(OH)D, depending on fish type [22]. Dietary sources therefore matter, but their effect on status cannot be inferred from intake data alone.

Serum data illustrate this caution. The Research on Osteoarthritis/Osteoporosis Against Disability (ROAD) surveys reported higher mean serum 25(OH)D in 2015-2016 than in 2005-2007 (25.1 versus 23.3 ng/mL) and a lower proportion below 20 ng/mL (21.6% versus 29.5%) [23]. Non-dietary determinants, including UV exposure and supplement use, may also contribute to differences in serum 25(OH)D. These surveys predated the 2019-2023 comparison in this study and differed in collection periods and participant composition, so lower dietary vitamin D intake should not be assumed to indicate lower serum 25(OH)D. Studies of selected populations show that prevalence estimates depend on the setting and threshold. Tsugawa and colleagues found high proportions below 20 ng/mL in a small study of young women [4], and Miyamoto and colleagues reported that 98% of 5,518 urban health-checkup attendees had concentrations below 30 ng/mL; a corrigendum has been published [5,6]. Neither sample represents all Japanese adults, and the thresholds used to define low status are not uniformly endorsed [7,8].

Eggs and mushrooms made relatively small contributions to vitamin D in the 2019 and 2023 food-group tables, indicating that current diets provide limited non-fish sources of this nutrient. UV-exposed mushrooms can contain increased vitamin D2, although this possibility was not evaluated in the present data [24]. In Finland, population-wide food fortification was associated with improved serum 25(OH)D in a representative adult sample [25], and food-fortification guidance recommends tailoring strategies to population intake and vitamin D status [26]. These findings support research on alternative sources of vitamin D in Japan but do not establish that fortification would have the same effects or be appropriate there.

Changes in fish consumption also matter for nutrients beyond vitamin D. Japanese survey analyses have reported lower fish and shellfish intake alongside higher meat intake [27]. From 2003 to 2019, adherence to a Japanese-style dietary pattern declined, while a bread-and-dairy pattern became more common and a meat-and-oil pattern increased among men [28]. Meat may also provide less eicosapentaenoic acid (EPA) and docosahexaenoic acid (DHA) than some fish species, although the NHNS food-group tables used here did not quantify these fatty acids. In a modeling study of Japanese women aged 20-39 years, estimated DHA intake and methylmercury exposure both fell with declining seafood consumption during 2011-2019; infant neurodevelopmental outcomes in that study were modeled, not directly measured [29]. The net effects of lower seafood intake therefore depend on fish species, replacement foods, and contaminants.

Economic and practical factors may also influence seafood consumption. The FY2025 White Paper on Fisheries reports that, among households of two or more persons, purchases of fresh fish and shellfish decreased by 2% in 2025, while annual expenditure increased by 1% [3]. It also identifies economy and convenience among major consumer food preferences and documents a long-term rise in preference for convenience [3]. These observations provide context but postdate the principal 2019-2023 dietary comparison and cannot be used to explain the differences observed in this study. The NHNS aggregate tables analyzed here do not include food prices, household expenditure, or reasons for food selection. Future studies linking dietary and economic data could examine the potential roles of affordability, convenience, and availability.

This study has several strengths. It uses national survey estimates spanning nearly a decade, restricts principal comparisons to surveys using matching food composition tables, and compares vitamin D, vitamin B12, and protein in parallel. This three-nutrient comparison highlights differences in dependence on fish and shellfish.

Several limitations should be considered. Published one-day intake estimates do not establish habitual intake or individual food substitutions. Individual dietary records, age- and sex-standardized estimates, survey-design variance estimates, measured serum 25(OH)D, and supplement-specific vitamin D intake were unavailable for this analysis. Changes in food composition tables, survey sampling, and weighting limit cross-year comparability; no surveys were conducted in 2020-2021. Fish-derived percentages are ratios of group means rather than mean individual percentages. Calculated residuals and amounts per 100 g are sensitive to rounding, and the 0.1-μg/day differences in calculated contributions from other foods are comparable to the uncertainty introduced by rounding and should not be interpreted as meaningful decreases. Extraction by a single author also introduces a potential risk of transcription error. For reference, the Dietary Reference Intakes for Japanese (2025 Edition) specify an adequate intake (AI) of 9.0 μg/day for adults [30]. The 2023 NHNS median one-day vitamin D intake was 3.1 μg/day, approximately one-third of the AI. However, intake below the AI does not necessarily indicate inadequacy, and these one-day data cannot estimate the prevalence of inadequate habitual intake.

Further research should use individual dietary data, survey weights, and harmonized composition tables to investigate which foods may replace fish and shellfish. Studies should examine habitual intake, age and sex differences, and associations with serum 25(OH)D while accounting for season, UV exposure, and supplements. Routine reporting of food-source contributions in national surveys could help detect nutrients whose supply depends heavily on a single food group. Models of replacement or fortification strategies should consider vitamin D, vitamin B12, long-chain n-3 fatty acids, cost, and methylmercury exposure.

## Conclusions

Fish and shellfish consistently supplied approximately three-quarters of the mean dietary vitamin D among Japanese adults in national surveys with adult-specific food-source data from 2016 to 2024. In the comparable 2019 and 2023 surveys, contributions of vitamin D and vitamin B12 from fish and shellfish were lower, whereas total protein intake changed little. Published mean contributions from other foods showed no apparent increase at the reported precision, although small differences were sensitive to rounding. These aggregate, one-day dietary estimates cannot establish individual food substitutions, habitual intake adequacy, or changes in serum 25(OH)D, and supplement-specific vitamin D intake was not assessed. The contrasting patterns across vitamin D, vitamin B12, and protein highlight the value of monitoring nutrient-specific food sources rather than relying on total protein intake alone.

## Appendices

**Table 3:** Source Tables for Values Extracted From the NHNS Reports Location: where the value appears in the manuscript. Values are reproduced exactly as reported in the manuscript and were used as published, at their reported precision. Table numbers refer to the printed National Health and Nutrition Survey (NHNS) report of each year [10-17]; the 2017 report numbers the food-group nutrient table as Table 9 rather than Table 9-2. "All other foods" is the combined total of all food groups other than fish and shellfish. "Calculated" entries were derived by the author from published rounded means; all other entries were transcribed from the source tables. Survey overview: the methods section of each report (sampling design and nutrient calculation). The 2015 food-group nutrient table covered people aged one year or older and was not used for adult-specific comparisons. -: not applicable; SD: standard deviation

No.	Year	Location	Variable	Value	Unit	Source table	Verification basis
A. Values in Table 1 (survey estimates, 2015-2024)							
1	2015	Table 1	Survey type	Routine	-	Survey overview	Sampling design: routine survey (300 districts)
2	2015	Table 1	Food composition table	2010	-	Survey overview	Nutrient calculation: food composition table edition
3	2015	Table 1	Sample size (n)	6,172	persons	Table 1-1	Adult column (≥20 years); PDF page image checked
4	2015	Table 1	Vitamin D, mean ± SD	7.9 ± 9.0	μg/day	Table 1-1	Adult column (≥20 years); PDF page image checked
5	2015	Table 1	Vitamin D, median	4.5	μg/day	Table 1-1	Adult column (≥20 years); PDF page image checked
6	2015	Table 1	Fish and shellfish, mean ± SD	74.2 ± 71.4	g/day	Table 5	Adult column (≥20 years); PDF page image checked
7	2015	Table 1	Fish and shellfish, median	60.8	g/day	Table 5	Adult column (≥20 years); PDF page image checked
8	2015	Table 1	Vitamin D from fish and shellfish	-	-	Not used	Not calculated: 2015 table covers ages ≥1 year
9	2016	Table 1	Survey type	Expanded	-	Survey overview	Sampling design: expanded (prefecture-level sampling)
10	2016	Table 1	Food composition table	2010	-	Survey overview	Nutrient calculation: food composition table edition
11	2016	Table 1	Sample size (n)	21,851	persons	Table 1-1	Adult column (≥20 years); PDF page image checked
12	2016	Table 1	Vitamin D, mean ± SD	7.8 ± 8.2	μg/day	Table 1-1	Adult column (≥20 years); PDF page image checked
13	2016	Table 1	Vitamin D, median	4.1	μg/day	Table 1-1	Adult column (≥20 years); PDF page image checked
14	2016	Table 1	Fish and shellfish, mean ± SD	70.5 ± 65.2	g/day	Table 5	Adult column (≥20 years); PDF page image checked
15	2016	Table 1	Fish and shellfish, median	58.0	g/day	Table 5	Adult column (≥20 years); PDF page image checked
16	2016	Table 1	Vitamin D from fish and shellfish	78	%	Table 9-2	Calculated: 6.1/7.8 × 100 = 78.205%, rounded to 78%
17	2017	Table 1	Survey type	Routine	-	Survey overview	Sampling design: routine survey (300 districts)
18	2017	Table 1	Food composition table	2010	-	Survey overview	Nutrient calculation: food composition table edition
19	2017	Table 1	Sample size (n)	5,794	persons	Table 1-1	Adult column (≥20 years); PDF page image checked
20	2017	Table 1	Vitamin D, mean ± SD	7.3 ± 8.2	μg/day	Table 1-1	Adult column (≥20 years); PDF page image checked
21	2017	Table 1	Vitamin D, median	4.0	μg/day	Table 1-1	Adult column (≥20 years); PDF page image checked
22	2017	Table 1	Fish and shellfish, mean ± SD	68.8 ± 69.2	g/day	Table 5	Adult column (≥20 years); PDF page image checked
23	2017	Table 1	Fish and shellfish, median	55.0	g/day	Table 5	Adult column (≥20 years); PDF page image checked
24	2017	Table 1	Vitamin D from fish and shellfish	75	%	Table 9	Calculated: 5.5/7.3 × 100 = 75.342%, rounded to 75%
25	2018	Table 1	Survey type	Routine	-	Survey overview	Sampling design: routine survey (300 districts)
26	2018	Table 1	Food composition table	2015 + S16-17	-	Survey overview	Nutrient calculation: food composition table edition
27	2018	Table 1	Sample size (n)	5,743	persons	Table 1-1	Adult column (≥20 years); PDF page image checked
28	2018	Table 1	Vitamin D, mean ± SD	7.0 ± 8.1	μg/day	Table 1-1	Adult column (≥20 years); PDF page image checked
29	2018	Table 1	Vitamin D, median	3.8	μg/day	Table 1-1	Adult column (≥20 years); PDF page image checked
30	2018	Table 1	Fish and shellfish, mean ± SD	70.1 ± 68.8	g/day	Table 5	Adult column (≥20 years); PDF page image checked
31	2018	Table 1	Fish and shellfish, median	59.0	g/day	Table 5	Adult column (≥20 years); PDF page image checked
32	2018	Table 1	Vitamin D from fish and shellfish	78	%	Table 9-2	Calculated: 5.4/6.9 × 100 = 78.261%, rounded to 78%
33	2019	Table 1	Survey type	Routine	-	Survey overview	Sampling design: routine survey (300 districts)
34	2019	Table 1	Food composition table	2015 + S16-18	-	Survey overview	Nutrient calculation: food composition table edition
35	2019	Table 1	Sample size (n)	4,927	persons	Table 1-1	Adult column (≥20 years); PDF page image checked
36	2019	Table 1	Vitamin D, mean ± SD	7.2 ± 8.9	μg/day	Table 1-1	Adult column (≥20 years); PDF page image checked
37	2019	Table 1	Vitamin D, median	3.6	μg/day	Table 1-1	Adult column (≥20 years); PDF page image checked
38	2019	Table 1	Fish and shellfish, mean ± SD	68.5 ± 71.5	g/day	Table 5	Adult column (≥20 years); PDF page image checked
39	2019	Table 1	Fish and shellfish, median	55.0	g/day	Table 5	Adult column (≥20 years); PDF page image checked
40	2019	Table 1	Vitamin D from fish and shellfish	78	%	Table 9-2	Calculated: 5.6/7.2 × 100 = 77.778%, rounded to 78%
41	2022	Table 1	Survey type	Routine	-	Survey overview	Sampling design: routine survey (300 districts)
42	2022	Table 1	Food composition table	2015 + S16-18	-	Survey overview	Nutrient calculation: food composition table edition
43	2022	Table 1	Sample size (n)	4,453	persons	Table 1-1	Adult column (≥20 years); PDF page image checked
44	2022	Table 1	Vitamin D, mean ± SD	6.5 ± 8.0	μg/day	Table 1-1	Adult column (≥20 years); PDF page image checked
45	2022	Table 1	Vitamin D, median	3.2	μg/day	Table 1-1	Adult column (≥20 years); PDF page image checked
46	2022	Table 1	Fish and shellfish, mean ± SD	61.4 ± 67.7	g/day	Table 5	Adult column (≥20 years); PDF page image checked
47	2022	Table 1	Fish and shellfish, median	42.8	g/day	Table 5	Adult column (≥20 years); PDF page image checked
48	2022	Table 1	Vitamin D from fish and shellfish	75	%	Table 9-2	Calculated: 4.9/6.5 × 100 = 75.385%, rounded to 75%
49	2023	Table 1	Survey type	Routine	-	Survey overview	Sampling design: routine survey (300 districts)
50	2023	Table 1	Food composition table	2015 + S16-18	-	Survey overview	Nutrient calculation: food composition table edition
51	2023	Table 1	Sample size (n)	4,472	persons	Table 1-1	Adult column (≥20 years); PDF page image checked
52	2023	Table 1	Vitamin D, mean ± SD	6.5 ± 7.9	μg/day	Table 1-1	Adult column (≥20 years); PDF page image checked
53	2023	Table 1	Vitamin D, median	3.1	μg/day	Table 1-1	Adult column (≥20 years); PDF page image checked
54	2023	Table 1	Fish and shellfish, mean ± SD	62.6 ± 67.8	g/day	Table 5	Adult column (≥20 years); PDF page image checked
55	2023	Table 1	Fish and shellfish, median	45.7	g/day	Table 5	Adult column (≥20 years); PDF page image checked
56	2023	Table 1	Vitamin D from fish and shellfish	77	%	Table 9-2	Calculated: 5.0/6.5 × 100 = 76.923%, rounded to 77%
57	2024	Table 1	Survey type	Expanded	-	Survey overview	Sampling design: expanded (prefecture-level sampling)
58	2024	Table 1	Food composition table	2015 + S16-18	-	Survey overview	Nutrient calculation: food composition table edition
59	2024	Table 1	Sample size (n)	15,716	persons	Table 1-1	Adult column (≥20 years); PDF page image checked
60	2024	Table 1	Vitamin D, mean ± SD	6.9 ± 6.5	μg/day	Table 1-1	Adult column (≥20 years); PDF page image checked
61	2024	Table 1	Vitamin D, median	3.3	μg/day	Table 1-1	Adult column (≥20 years); PDF page image checked
62	2024	Table 1	Fish and shellfish, mean ± SD	63.0 ± 51.2	g/day	Table 5	Adult column (≥20 years); PDF page image checked
63	2024	Table 1	Fish and shellfish, median	46.7	g/day	Table 5	Adult column (≥20 years); PDF page image checked
64	2024	Table 1	Vitamin D from fish and shellfish	77	%	Table 9-2	Calculated: 5.3/6.9 × 100 = 76.812%, rounded to 77%
B. Values in Table 2 (food-source contributions, 2019 and 2023)							
65	2019	Table 2	Vitamin D, total	7.2	μg/day	Table 9-2	Adult (≥20 years) row; nutrient column
66	2023	Table 2	Vitamin D, total	6.5	μg/day	Table 9-2	Adult (≥20 years) row; nutrient column
67	2019	Table 2	Vitamin D, fish and shellfish	5.6	μg/day	Table 9-2	Adult (≥20 years) row; nutrient column
68	2023	Table 2	Vitamin D, fish and shellfish	5.0	μg/day	Table 9-2	Adult (≥20 years) row; nutrient column
69	2019	Table 2	Vitamin D, all other foods	1.6	μg/day	Table 9-2	Calculated: total - fish and shellfish (both to 0.1)
70	2023	Table 2	Vitamin D, all other foods	1.5	μg/day	Table 9-2	Calculated: total - fish and shellfish (both to 0.1)
71	2019	Table 2	Vitamin D, of which meat	0.2	μg/day	Table 9-2	Adult (≥20 years) row; nutrient column
72	2023	Table 2	Vitamin D, of which meat	0.2	μg/day	Table 9-2	Adult (≥20 years) row; nutrient column
73	2019	Table 2	Vitamin D, of which eggs	0.7	μg/day	Table 9-2	Adult (≥20 years) row; nutrient column
74	2023	Table 2	Vitamin D, of which eggs	0.7	μg/day	Table 9-2	Adult (≥20 years) row; nutrient column
75	2019	Table 2	Vitamin D, of which mushrooms	0.2	μg/day	Table 9-2	Adult (≥20 years) row; nutrient column
76	2023	Table 2	Vitamin D, of which mushrooms	0.2	μg/day	Table 9-2	Adult (≥20 years) row; nutrient column
77	2019	Table 2	Vitamin B12, total	6.5	μg/day	Table 9-2	Adult (≥20 years) row; nutrient column
78	2023	Table 2	Vitamin B12, total	5.5	μg/day	Table 9-2	Adult (≥20 years) row; nutrient column
79	2019	Table 2	Vitamin B12, fish and shellfish	4.4	μg/day	Table 9-2	Adult (≥20 years) row; nutrient column
80	2023	Table 2	Vitamin B12, fish and shellfish	3.5	μg/day	Table 9-2	Adult (≥20 years) row; nutrient column
81	2019	Table 2	Vitamin B12, all other foods	2.1	μg/day	Table 9-2	Calculated: total - fish and shellfish (both to 0.1)
82	2023	Table 2	Vitamin B12, all other foods	2.0	μg/day	Table 9-2	Calculated: total - fish and shellfish (both to 0.1)
83	2019	Table 2	Vitamin B12, of which meat	0.9	μg/day	Table 9-2	Adult (≥20 years) row; nutrient column
84	2023	Table 2	Vitamin B12, of which meat	0.8	μg/day	Table 9-2	Adult (≥20 years) row; nutrient column
85	2019	Table 2	Protein, total	72.2	g/day	Table 9-2	Adult (≥20 years) row; nutrient column
86	2023	Table 2	Protein, total	71.3	g/day	Table 9-2	Adult (≥20 years) row; nutrient column
87	2019	Table 2	Protein, fish and shellfish	13.1	g/day	Table 9-2	Adult (≥20 years) row; nutrient column
88	2023	Table 2	Protein, fish and shellfish	12.0	g/day	Table 9-2	Adult (≥20 years) row; nutrient column
89	2019	Table 2	Protein, all other foods	59.1	g/day	Table 9-2	Calculated: total - fish and shellfish (both to 0.1)
90	2023	Table 2	Protein, all other foods	59.3	g/day	Table 9-2	Calculated: total - fish and shellfish (both to 0.1)
91	2019	Table 2	Protein, of which meat	17.3	g/day	Table 9-2	Adult (≥20 years) row; nutrient column
92	2023	Table 2	Protein, of which meat	18.2	g/day	Table 9-2	Adult (≥20 years) row; nutrient column
C. Values cited only in the Results text							
93	2023	Results	Vitamin D, median (20-29 years)	2.1	μg/day	Table 1-1	Age-group columns (20-29 and ≥75 years)
94	2023	Results	Vitamin D, median (≥75 years)	4.2	μg/day	Table 1-1	Age-group columns (20-29 and ≥75 years)
95	2023	Results	Fish and shellfish, median (20-29 years)	15.0	g/day	Table 5	Age-group columns (20-29 and ≥75 years)
96	2023	Results	Fish and shellfish, median (≥75 years)	68.7	g/day	Table 5	Age-group columns (20-29 and ≥75 years)
97	2019	Results	Meat intake, mean	101.0	g/day	Table 9-2	Adult (≥20 years) row
